# Time-Dependent Open-Quantum Approach to Two-Dimensional Electronic Spectroscopy within a GW/BSE Active Space

**DOI:** 10.1021/acs.jctc.5c02002

**Published:** 2026-02-25

**Authors:** Giulia Dall’Osto, Margherita Marsili, Stefano Corni, Emanuele Coccia

**Affiliations:** † Dipartimento di Scienze Chimiche e Farmaceutiche, 9315 University of Trieste, via L. Giorgieri 1, 34127 Trieste, Italy; ‡ Dipartimento di Fisica e Astronomia ”Augusto Righi”, University of Bologna, Viale Berti Pichat 6/2, 40127 Bologna, Italy; § Dipartimento di Scienze Chimiche, Università di Padova, via F. Marzolo 1, 35131 Padova, Italy; ∥ Istituto Nanoscienze-CNR, via Campi 213/A, 41125 Modena, Italy

## Abstract

In this work, we present a theoretical and computational approach that combines real-time propagation of the electronic wave function, the GW/BSE formalism for the electronic structure of ground and excited states, the theory of open quantum systems, and the phase-cycling method to compute two-dimensional electronic spectra (2DES) of molecular systems under realistic excitation conditions. The advantage of this strategy is that it combines the accuracy of first-principle calculations such as GW/BSE with an explicit description of the employed laser pulses. This allows for better adherence to experimental setups. We apply the proposed methodology to benzene, chlorophyll *b*, and *a* benzene–phenol dimer, also including a pure electronic dephasing in the time propagation. The calculated 2DES maps reveal clear signatures of stimulated emission and excited-state absorption, as well as coherence dynamics as a function of the population time, both in the absence and presence of pure dephasing. Comparison with experimental and theoretical published data has been carried out, when available.

## Graphical abstract

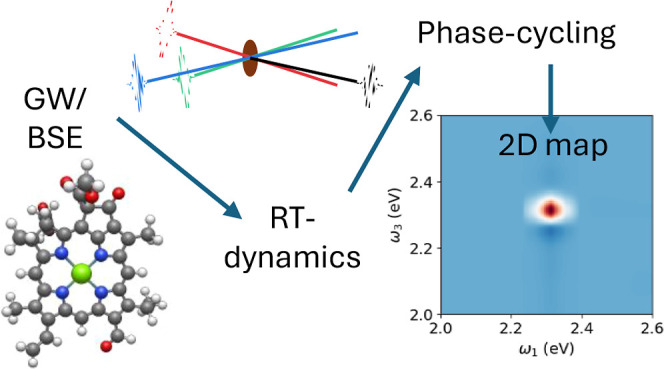

## 1 Introduction

Two-dimensional electronic spectroscopy (2DES)^1–3^ is a nonlinear optical technique that involves the interaction of a sample with a sequence of ultrafast pulses, resulting in a map that correlates the excitation and the detection frequencies.^4^ After the interaction of the sample with three (noncollinear) laser pulses, each separated by a precisely controlled delay time, the third-order polarization of the system is collected in a phase-matching direction. Two-dimensional Fourier transform of the nonlinear signal with respect to the delay time between the first two pulses and the detection time, gives the 2DES spectrum at a specific population time (e.g., the delay time between the second and the third pulse).

2DES has proven to be an exceptional tool for probing quantum coherence and energy transfer pathways in light-harvesting systems central to photosynthetic processes.^2,5–8^ Nonlinear techniques were able to demonstrate that excitation of multichromophoric systems exhibit coherent wave-like energy transfer, suggesting that quantum effects may enhance the efficiency of photosynthesis.^7–10^ Over time, its applications have expanded to encompass a wide range of systems, including organic multichromophore complexes^11–13^ and fully inorganic materials,^14,15^ with many potential applications still to be explored.

The wealth of information embedded in a 2DES spectrum necessitates the use of advanced theoretical tools for accurate interpretation.^16–20^ Within the formalism of the density matrix, Mukamel and co-workers have developed a unique and versatile approach for modeling multidimensional femtosecond spectroscopy.^21,22^ In the framework of perturbation theory, the density matrix is expanded in powers and the nonlinear response is computed as the expectation value of the dipole operator on the perturbative density matrix.^23–25^ In many cases, the system evolution is performed assuming external perturbations under the impulsive limit. The density matrix formalism has also been coupled with ab initio calculations of the systems under study.^26,27^ Accurate results have been provided by using quantum mechanics/molecular mechanics scheme introducing the effect of the environment.^28–30^

Moving from perturbation theory, a different approach for extracting the nonlinear response, particularly in the context of four-wave mixing interactions, is based on a linear combination scheme of Liouville pathways, where interactions are modeled as complex pulses.^31,32^ This method, also known as phase-matching approach, relies on a combination of a fixed number of independent density matrix propagations.^33^ In some cases, when conical intersections became relevant in the system dynamics, nonadiabatic coupling is also included, for instance, with trajectory surface-hopping approaches.^34,35^ Also, a combination of molecular dynamics, exciton representation of the complex system, and quantum chemistry has been successfully employed to compute four-wave mixing signals.^18^

Another strategy has been developed involving the wave function propagation, where nonlinear polarization is obtained combining wave functions evolved in the bra and ket spaces including an opportune number of interactions with pulses in each space.^36^ Wave function methods have proven to be a valid alternative, not only allowing for results comparable to density matrix dynamics but also offering complementary understanding.^37^ To better align the experiment with theory, the system wave function is propagated using a real field with a finite temporal duration, including the pulse overlap effects. From these simulations, the expectation value of the dipole moment gives the overall polarization dynamics, so an additional procedure such as the phase-cycling scheme is needed to extract the third-order signal along a phase-matching direction.^20,38,39^

Our aim is to develop a framework where an accurate yet affordable ab initio description is coupled to a real-time representation of the system dynamics that allows treating external electromagnetic pulses of arbitrary time-profile and include also decoherence effects. To this end, we employ the GW/Bethe–Salpeter equation (GW/BSE) approach, which is well established for its accurate description of quasiparticle band gaps and its ability to properly capture charge-transfer excitations and neutral excitation energies, including excitonic effects, in molecular systems.^40,41^ Within this many-body perturbation theory framework, the DFT electronic structure is improved by incorporating dynamical electronic correlation through a self-energy term expressed as the product of the single-particle Green’s function *G* and the screened Coulomb interaction *W*. On top of this corrected electronic structure, neutral excitation energies and the corresponding transition dipole moments are obtained by solving the BSE, which explicitly includes electron–hole interactions. Using GW/BSE allows one to achieve high accuracy, such as that by coupled cluster methods, with a much smaller computational effort, thus making the investigation of larger molecular and composite systems feasible. Recently, a GW/BSE-derived electronic active space has been exploited within real-time wave function propagation schemes that explicitly account for the interaction with external electric fields.^42,43^

In the present framework, the system is modeled as both closed whose real-time propagation is carried out using the coherent time-dependent Schrödinger equation (TDSE), and as open, when the stochastic Schrödinger equation (SSE)^44^ is used to propagate the system wave function in the presence of dephasing or relaxation channels.^45–50^

The methodological novelty of the present approach lies in the seamless integration of these elements into a single, fully ab initio, open-quantum real-time framework for 2DES simulations. Specifically, we combine (i) a GW/BSE-based electronic structure and excitation manifold, (ii) explicit real-time propagation driven by shaped ultrafast laser pulses of (generally) any shape, and (iii) an open-quantum-system description via the SSE.

The proposed approach has been applied to compute the 2DES spectra of two molecular systems, i.e., benzene and the porphyrin core of chlorophyll *b*,^51^ a representative case of a relevant chromophore that has biological interest due to its involvement in photosynthetic processes. Furthermore, a benzene-phenol dimer has been considered as a representation of the lateral chain of two amino acids at a sufficient large distance to avoid the charge transfer mechanism, as proposed in ref 30. In this case, the focus is on getting information on coherence dynamics as a function of the population time. For this reason, we have combined the GW/BSE-based dynamics with the SSE to simulate environment-induced electronic decoherence. These systems were chosen to validate our approach, possibly comparing our results with data available in the literature.

In Section 2, the proposed theoretical method is detailed; computational details of the simulations are collected in Section 3. Results are shown and discussed in Section 4, while conclusions and future perspectives are summarized in Section 5.

## 2 Theory

The workflow for computing the 2DES spectra is illustrated in Figure 1. It begins with quantum-mechanical calculations at the GW/BSE level of theory, which provide the excitation energies and transition dipole moments of the target system (Section 2.1). These quantities serve as the input for the real-time propagation of the system wave function. The propagation is performed using either TDSE or the SSE, depending on whether the system is treated as closed or open (Section 2.2). The nonlinear polarization along a specific direction is extracted using the phase-cycling technique (Section 2.3), and the 2DES spectrum is obtained through a double Fourier transform (Section 2.4). This section outlines each of the steps involved in computing the simulated 2DES spectrum.

**Figure 1:**
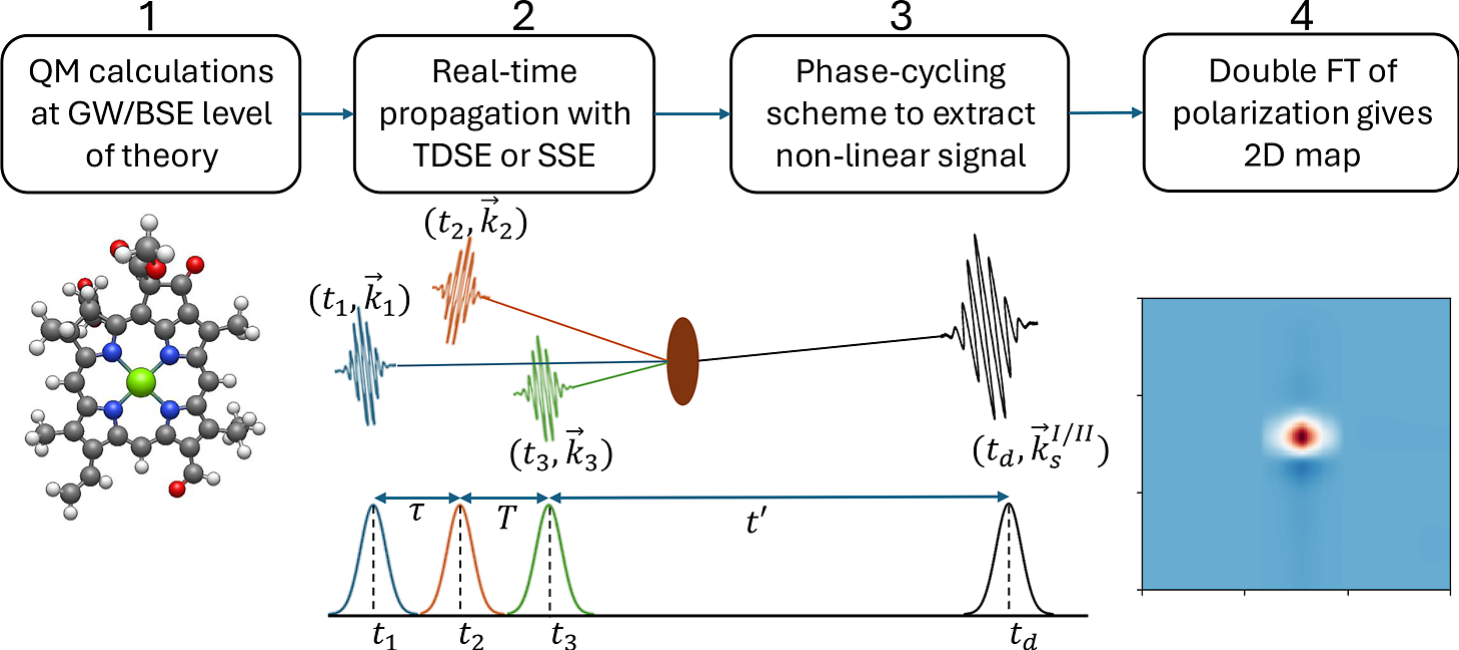
Workflow scheme for computing 2DES spectra used in this work.

### 2.1 GW/BSE Electronic Active Space

The real-time dynamics (Section 2.2) is carried out within a multielectronic active space derived within the GW/BSE approach^40^ as introduced in ref 43. Here, we briefly recall its main features. Within the GW/BSE approach, building on top of an accurate determination of the electronic energy levels, i.e. the GW-corrected Kohn–Sham eigenvalues, the optical spectra are obtained diagonalizing an effective two-particle Hamiltonian that describes the correlated motion of an electron/hole couple within the system. In an optical spectrum computed at an independent-particle level, every single-particle transition contributes independently to the others, with a weight given by its transition dipole moment at the energy of the single-particle transition itself. In contrast, in the GW/BSE spectra, structures are located at specific energies, obtained from the diagonalization of the effective two-particle Hamiltonian. The weight of each structure is given by linear combinations of single-particle transition dipole moments, whose coefficients are the components of the corresponding two-particle Hamiltonian eigenvectors.

The GW/BSE active space ansatz is the following

1 $$\{\begin{array}{l} {Ĥ}_{0}|\lambda \rangle =E_{\lambda }|\lambda \rangle \\ |\lambda \rangle =\sum_{cv}A_{cv}^{\lambda }a_{c}^{†}a_{v}|0\rangle , \end{array}$$

where the indices *c* and *v* run over unoccupied and occupied molecular orbitals respectively with the corresponding creation, *a*_c_^†^, and annihilation, *a*_v_ operators. *E*_λ_ and *A*_cv_^λ^ are the eigenvalues and eigenfunctions of the BSE effective two-particle Hamiltonian in its resonant part. ${Ĥ}_{0}$ is the field-free electronic Hamiltonian. The GW/BSE active space includes the ground state |0⟩ and a set of excited states {|λ⟩} obtained by solving the GW/BSE equation. The {|λ⟩} states are indeed linear combinations of singly excited Slater determinants, whose coefficients are determined through the solution of the BSE equation. Slater determinants are built considering the number of occupied and virtual molecular orbitals selected for GW/BSE calculations. This choice implies the use of all occupied orbitals within the frozen-core assumption, and a large number of virtual ones guaranteeing convergence, as reported in Section 3. From the knowledge of the {λ} states, the matrix elements of any operator can be obtained, as, e.g., the transition dipole moments $\left\langle \lambda^{\prime }\left|\mathrm{\mu ⃗}\right|\lambda \right\rangle$ that are needed to couple the electronic system with the explicit external field, as discussed in Section 2.2.

### 2.2 Real-Time Propagation

Real-time photoinduced processes are simulated by the TDSE, that is given in length gauge by^52,53^

2 $$i\frac{d}{dt}|\Psi \left(t\right)\rangle =Ĥ\left(t\right)|\Psi \left(t\right)\rangle$$

where |Ψ­(*t*)⟩ is the time-dependent wave function, and the time-dependent Hamiltonian *Ĥ*(*t*) is defined as

3 $$Ĥ\left(t\right)={Ĥ}_{0}-\vec{\hat{\mu }}\cdot \mathrm{F⃗}\left(t\right)$$

with ${Ĥ}_{0}$ and $\vec{\hat{\mu }}$ being the field-free electronic Hamiltonian of eq 1 and the dipole of the target respectively, and F⃗(t) the external field. The incident radiation is a sum of three laser pulses with identical shape, modeled as^38,39^

4 $$\mathrm{F⃗}\left(t\right)=\sum_{i=1}^{3}{\mathrm{F⃗}}_{i,0}\;\cos\left(\omega \left(t-t_{i}\right)-\phi_{i}\right)e^{-{\left(t-t_{i}\right)}^{2}/2\delta^{2}}$$

where *t*_*i*_ is the center of each pulse, ω (the same for each pulse) and ϕ_*i*_ are the frequency and the phase, ${\mathrm{F⃗}}_{i,0}$ is the amplitude vector and δ is the pulse width. Atomic units are used throughout the article.

The time-dependent wave function |Ψ­(*t*)⟩ is represented as a linear combination of electronic states, that are eigenstates of ${Ĥ}_{0}$

5 $$|\Psi \left(t\right)\rangle =\sum_{\lambda =0}^{N_{states}-1}C_{\lambda }\left(t\right)|\lambda \rangle$$

with *C*_λ_(*t*) being time-dependent coefficients, and |λ⟩ is an eigenstate of the target system. In the space of the ${Ĥ}_{0}$ eigenstates {|λ⟩}, the TDSE is rewritten as

6 $$i\frac{dC\left(t\right)}{dt}=H\left(t\right)C\left(t\right)$$

where **C**(*t*) is the vector of the expansion coefficients and **H**(*t*) is the matrix representation at time *t* of the time-dependent Hamiltonian. The matrix element (**H**(*t*))_λ′,λ_ is explicitly given by

7 $${\left(H\left(t\right)\right)}_{\lambda^{\prime },\lambda }=E_{\lambda }\delta_{\lambda^{\prime },\lambda }-\mathrm{F⃗}\left(t\right)\cdot \left\langle \lambda^{\prime }\left|\vec{\hat{\mu }}\right|\lambda \right\rangle$$

where *E*_λ_ (eq 1) and $\left\langle \lambda^{\prime }\left|\vec{\hat{\mu }}\right|\lambda \right\rangle$ are the excitation energies and the transition dipole moments obtained by the GW/BSE calculation and used as input for the real-time dynamics. Details on how $\left\langle \lambda^{\prime }\left|\vec{\hat{\mu }}\right|\lambda \right\rangle$ are computed within this ansatz are found in ref 43.

Equation 6 is propagated according to a second-order Euler algorithm^45^

8 C(t+δt)=C(t−δt)−2i⁡δt⁡H(t)⁡C(t)

with δ*t* being the discrete time step for TDSE propagation.

#### 2.2.1 Stochastic Schrödinger Equation

In the picture of open quantum systems,^54^ the effect of the environment on the system dynamics can be included by propagating the wave function with the time-dependent SSE.^44^ In the Markovian limit, the environment correlation function is a Dirac delta, due to the faster time scale of bath equilibration compared to the system dynamics. The Markovian time-dependent SSE^44^ is written as

9 $$i\frac{d}{dt}|\Psi_{sys}\left(t\right)\rangle ={Ĥ}_{sys}\left(t\right)|\Psi_{sys}\left(t\right)\rangle -\frac{i}{2}\sum_{q}^{M}{Ŝ}_{q}^{†}{Ŝ}_{q}|\Psi_{sys}\left(t\right)\rangle +\sum_{q}^{M}l_{q}\left(t\right){Ŝ}_{q}|\Psi_{sys}\left(t\right)\rangle$$

where |Ψ_sys_(*t*)⟩ and ${Ĥ}_{sys}\left(t\right)$ are the system wave function and the system Hamiltonian, respectively. |Ψ_sys_(*t*)⟩ and ${Ĥ}_{sys}\left(t\right)$ coincide with |Ψ­(*t*)⟩ and Ĥ(t) in the case of a closed-system TDSE. According to the fluctuation–dissipation theorem,^55^ the non-Hermitian term $-\frac{i}{2}\sum_{q}^{M}{Ŝ}_{q}^{†}{Ŝ}_{q}$ models the dissipation due to the environment, whereas $\sum_{q}^{M}l_{q}\left(t\right){Ŝ}_{q}$ is the fluctuation based on a Wiener process *l*_*q*_(*t*), i.e. a white noise associated with the Markov approximation. ${Ŝ}_{q}$ are operators describing the *q*-th of *M* interaction channels between the system and environment, such as nonradiative decay^56^ or pure dephasing.^45,48^ In our case, the interaction channels are represented in the basis {λ} of the wave function, thus the generic subscript *q* is substituted by λ. Moreover, the only open-system effect included in this work is the pure electron dephasing, indicated with the “dep” superscript $\left({Ŝ}_{\lambda }^{dep}\right)$ . The pure dephasing operator generates (electronic) decoherence, keeping the population untouched. It is defined for each λ electronic state as^45^

10 $${Ŝ}_{\lambda }^{dep}=\sqrt{\gamma_{\lambda }/2}\sum_{\lambda^{\prime }}D\left(\lambda ,\lambda^{\prime }\right)|\lambda^{\prime }\rangle \langle \lambda^{\prime }|$$

where *D*(λ, λ′) is defined as the following: it is equal to −1 if λ′ = λ or equal to 1 otherwise. Dephasing time *T*_2_ is equal to the inverse of γ_λ_, which is given as a phenomenological parameter. Since we use the same γ_λ_ per each state, an unique value of $T_{2}=\frac{1}{\gamma }$ with γ≡γ_λ_ is given.

Within the SSE framework, the density matrix of the system (e.g., the reduced density matrix obtained tracing out the bath degrees of freedom from the total density matrix) is practically recovered by averaging over a large number *N*_traj_ of quantum stochastic^44^ trajectories, namely

11 $${\mathrm{\rho ̂}}_{S}\left(t\right)=\frac{1}{N_{traj}}\sum_{j}^{N_{traj}}|\Psi_{S,j}\left(t\right)\rangle \langle \Psi_{S,j}\left(t\right)|$$

with |Ψ_*S*,*j*_(*t*)⟩ being the system wave function in *j*-th trajectory. Each |Ψ_*S*,*j*_(*t*)⟩ is expanded as in eq 5 according to the given level of theory used for the representation of the system. As a consequence, diagonal and off-diagonal elements of the reduced density matrix ${\mathrm{\rho ̂}}_{S}\left(t\right)$ , i.e. the populations and coherences of the states of the system at time *t*, respectively, are obtained by averaging on the number of independent realizations *N*_traj_ of propagating SSE.

SSE propagation is practically carried out by using a quantum jump algorithm^57–60^ to model the fluctuation term, based on Monte Carlo techniques,^61,62^ combined with a deterministic dissipative dynamics simulated by eq 8 in the presence of the non-Hermitian term $-\frac{i}{2}\sum_{q}^{M}{Ŝ}_{q}^{†}{Ŝ}_{q}$ . Details are given in our ref 45. TDSE and SSE are implemented in the WaveT code.^63^

### 2.3 Phase-Cycling Approach

The total polarization is computed as the expectation value of the transition dipole moment^21,22^

12 $$\mathrm{P⃗}\left(t\right)=\langle \Psi \left(t\right)|\hat{\vec{\mu }}|\Psi \left(t\right)\rangle =\sum_{\lambda^{\prime },\lambda }C_{\lambda^{\prime }}^{*}\left(t\right)C_{\lambda }\left(t\right)\langle \lambda^{\prime }|\vec{\hat{\mu }}|\lambda \rangle$$

Since no approximation has been introduced, eq 12 is the total polarization including all orders of a perturbative expansion. A further step is needed in order to decompose the total polarization into the component along different phase-matching directions. Therefore, a phase cycling scheme procedure is applied to extract the polarization along a specific direction. In this protocol,^39^ the dynamics is performed for 12 different combinations of phases of the three pulses ϕ_*s*_ = (ϕ_1_, ϕ_2_, ϕ_3_) (eq 4), each producing a phase dependent polarization. The total polarization is decomposed in a Fourier series^64^

13 $$\mathrm{P⃗}\left(t,\phi_{s}\right)=\sum_{l,m,n}{\mathrm{P⃗}}_{lmn}\left(t\right)e^{i\left(l\phi_{1}+m\phi_{2}+n\phi_{3}\right)}$$

in which each ${\mathrm{P⃗}}_{lmn}\left(t\right)$ term is the polarization along a phase-matching condition: ${\mathrm{k⃗}}_{s}=l{\mathrm{k⃗}}_{1}+m{\mathrm{k⃗}}_{2}+n{\mathrm{k⃗}}_{3}$ . In a four-wave mixing scheme,^65^ the signal can be detected along ${\mathrm{k⃗}}_{s}^{I}=-{\mathrm{k⃗}}_{1}+{\mathrm{k⃗}}_{2}+{\mathrm{k⃗}}_{3}$ direction, which gives the rephasing term and along ${\mathrm{k⃗}}_{s}^{II}={\mathrm{k⃗}}_{1}-{\mathrm{k⃗}}_{2}+{\mathrm{k⃗}}_{3}$ that is responsible for the nonrephasing term. Choosing different phase values and inserting the corresponding coefficients in the linear system, leads to the corresponding equations for the rephasing and nonrephasing terms^39^

14 $$\begin{array}{l} {\mathrm{P⃗}}_{-1,+1,+1}\left(t\right)=\sum_{s}c_{s}^{I}\mathrm{P⃗}\left(t,\phi_{s}\right),\\ {\mathrm{P⃗}}_{+1,-1,+1}\left(t\right)=\sum_{s}c_{s}^{II}\mathrm{P⃗}\left(t,\phi_{s}\right) \end{array}$$

The phase values and weights of the linear combination are reported in Table 1.^39^

**Table 1:** Phase Values Used in the Simulations and the Weights of Linear Combination of Phase Dependent Polarization Used to Compute ${\mathrm{P⃗}}_{-1,+1,+1}$ and ${\mathrm{P⃗}}_{+1,-1,+1}$

*s*	1	2	3	4	5	6	7	8	9	10	11	12
ϕ_1_	0	0	π/2	π/2	π	π	3π/2	0	0	π/2	3π/2	3π/2
ϕ_2_	0	0	0	0	0	0	0	0	0	0	0	0
ϕ_3_	0	π/2	π	π/2	0	π/2	3π/2	3π/2	π	3π/2	π	π/2
*c_s_^I^*	0	1- i	1 + *i*	–1	0	0	–1	1 + *i*	–2	-i	1-i	*i*
*c^_s_II^*	1 + *i*	–1- i	1-i	0	–1-i	1 + *i*	1-i	0	0	–1 + *i*	–1 + *i*	0

Other methods have been proposed in the literature to efficiently choose the minimum number of phase-dependent propagations needed to compute the nonlinear signal after the interaction with three pulses.^66,67^ In the present work we rely on the theory developed by Domcke and Engel who proposed 12 propagations to compute both rephasing and nonrephasing terms.^38,39,64^

### 2.4 Computing the Spectrum

The signal is computed as a function of the three delay times τ = *t*_2_ – *t*_1_, *T* = *t*_3_ – *t*_2_, *t*′ = *t*_*d*_ – *t*_3_, where *t*_*d*_ is the detection time (Figure 1). Although the polarization ${\mathrm{P⃗}}_{lmn}\left(t\right)$ already depends on the choice of τ and *T*, the dependence on *t*′ is obtained by changing the variable and shifting the time scale with respect to *t*_3_, i.e. setting *t*_3_ to 0 and considering the dynamics from this point until *t*_*d*_. After the time shift of the time-dependent polarization, the rephasing and nonrephasing polarization vectors as a function of the three delay times are defined as ${\mathrm{P⃗}}_{-1,+1,+1}\left(\tau ,T,t^{\prime }\right)$ and ${\mathrm{P⃗}}_{+1,-1,+1}\left(\tau ,T,t^{\prime }\right)$ . Which give respectively the rephasing *S*_R_(τ, *T*, *t*′) and nonrephasing signal *S*_NR_(τ, *T*, *t*′) assuming isotropic average.

Conventionally, the second delay time is kept in time domain while the final 2DES signal is obtained through a two-dimensional Fourier transform of the signal along the first and the third delay times. In case of the rephasing term (*S*_R_), the Fourier transform is forward with respect to the first delay time and backward with respect to the second, while in case of nonrephasing term (*S*_NR_) a double backward transform is performed.

15 $$\begin{array}{l} S_{R}\left(\omega_{1},T,\omega_{3}\right)=i\int_{0}^{\infty }d\tau \int_{0}^{\infty }dt^{\prime }e^{-i\omega \tau }e^{i\omega t^{\prime }}S_{R}\left(\tau ,T,t^{\prime }\right),\\ S_{NR}\left(\omega_{1},T,\omega_{3}\right)=i\int_{0}^{\infty }d\tau \int_{0}^{\infty }dt^{\prime }e^{i\omega \tau }e^{i\omega t^{\prime }}S_{NR}\left(\tau ,T,t^{\prime }\right). \end{array}$$

The frequencies ω_1_ and ω_3_ (Figure 1) respectively refer to the Fourier transform of the first and the third delay time. In many cases, the real part of the absorptive spectrum, defined as the sum of the rephasing and nonrephasing contributions, is reported, as it typically offers better resolution and is the quantity most commonly presented in experimental results.^65^

## 3 Computational Details

The ground-state optimized geometry of benzene was taken from ref 68. DFT and GW/BSE calculations were performed at CAM-B3LYP/aug-cc-pVTZ level of theory considering G3W3 correction as implemented in the MOLGW code.^69^ 100 virtual molecular orbitals have been included to build the single-particle Green’s function *G*, whereas 300 virtual molecular orbitals were used to build the RPA screening. The first 100 BSE excitation energies have been computed, 24 of which were used as {|λ⟩} set of states for the real-time dynamics. They were selected on the basis of transition dipole moments analysis: only excitations with a magnitude of the transition dipole moment greater than 0.02 au. Energies and ground-to excited-state transition dipole moments (though the full matrix has been used in all the calculations) have been reported in Table S1 of Supporting Information.

Ground-state geometry of the porphyrin core of chlorophyll *b* has been optimized at B3LYP/TZP level of theory using the ADF code within the AMS suite.^70^ A DFT and GW/BSE calculation has been carried out with MOLGW at CAM-B3LYP/cc-pVDZ level of theory, considering G3W3 corrections, including 500 virtual states to build *G* and 500 virtual states for W. The first 20 BSE eigenstates were used to build the set {|λ⟩}. Cartesian coordinates are provided in Table S2 of the Supporting Information. Energies and ground-to-excited-state transition dipole moments have been reported in Table S3 of the Supporting Information.

Optimized ground-state geometry of phenol was taken from ref 68. The benzene-phenol dimer was built using the molecular coordinates given in ref 30 placing the two molecules at 11.31 Å (center-of-mass distance) in a noninteracting scheme. Indeed, due to the large separation, the dimer geometry is simply the sum of the isolated molecules, individually optimized in ref 68. A DFT and GW/BSE calculation has been performed using MOLGW at CAM-B3LYP/cc-pVDZ level of theory, including G3W3 correction. 200 virtual molecular orbitals were used for *G* and 400 virtual molecular orbitals were used for *W* correction. The calculation of excitation energies and transition dipole moments has been performed in the ground-state geometry, including the first 150 BSE eigenvalues to cover the absorption spectrum from 4 to 9 eV. Energies and ground-to-excited transition dipole moments have been reported in Table S4 of the Supporting Information. Figures S1–S4 of the Supporting Information show the molecular orbital states involved in the most relevant transitions of the dimer.

All BSE calculations were performed applying the Tamm-Dancoff approximation. The structures of the molecules used in the calculations are reported in Figure 2.

**Figure 2:**
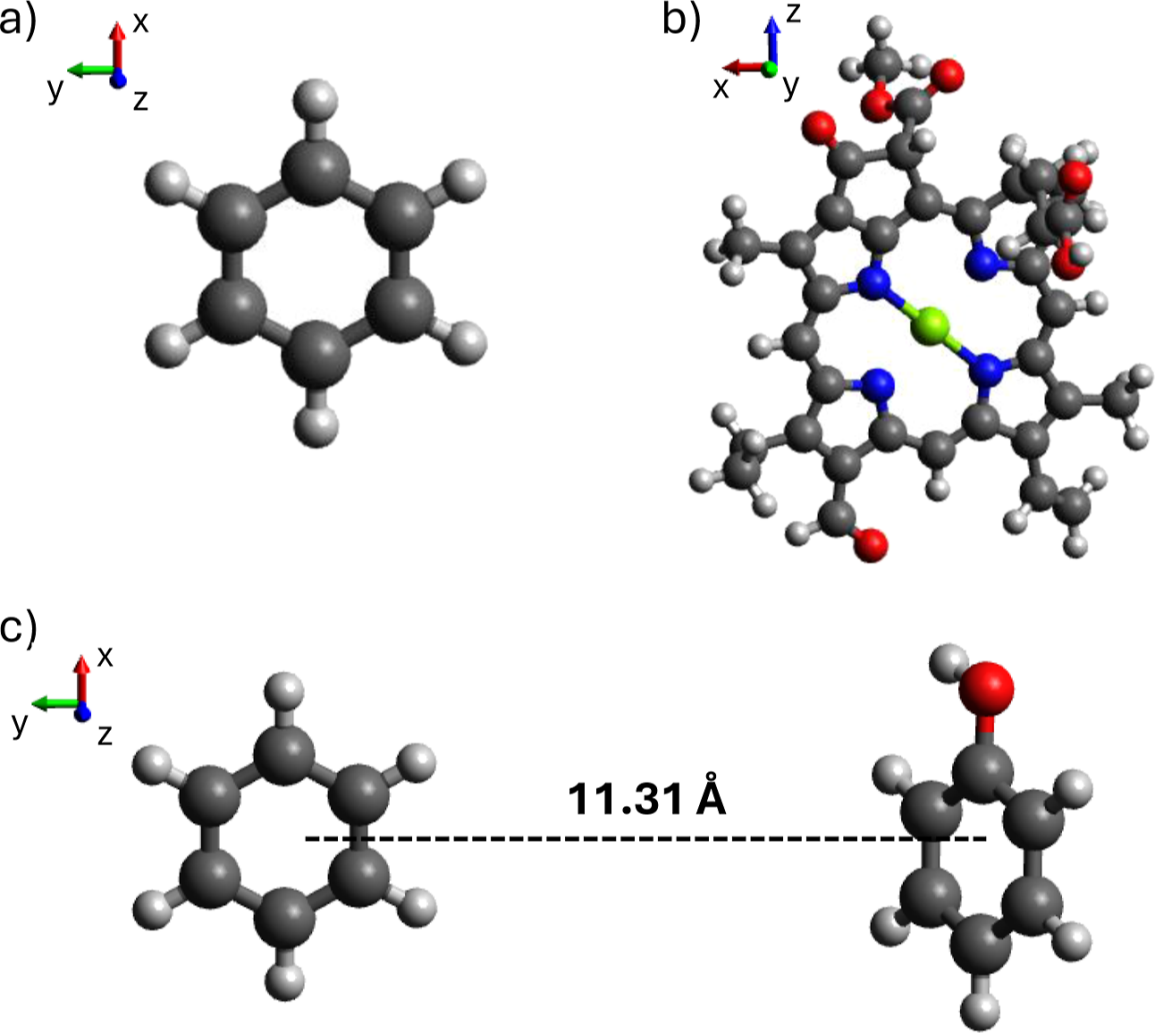
(a) Optimized geometry of benzene at CASSCF/ANO level taken from ref 68. (b) Porphyrin core of chlorophyll *b* optimized geometry at B3LYP/TZP level of theory. (c) Benzene-phenol dimer built from optimized geometry of the two molecules at CASSCF/ANO level, taken from Supporting Information of refs 30 and 68 . The distance between the molecular center of masses is 11.31 Å. Color correspondence: H: light gray, C: black, O: red, N: blue, Mg: green.

The real-time dynamics have been propagated for 484 fs (for benzene) or 1.2 ps (for chlorophyll *b* and the dimer) with 0.0242 fs time step. The delay time between the first and the second pulses has been scanned from 0 to 100 fs with a 0.5 fs step. The intensity of the pulses in all the simulations is equal to *I* = 5.01 × 10^9^ W/cm^2^. The other features of the electric field have been modified according to the system under study, and detailed in Section 4. To construct the 2DES spectrum, 12 propagations were performed for each value of τ, varying the phases of the three pulses according to Table 1. The (singlet) excited state is indicated with S_λ_.

## 4 Results and Discussion

The strategy explained above has been applied to obtain the 2DES spectra of benzene and of the porphyrin core of chlorophyll *b* at fixed population time. Afterward, we consider a noninteracting benzene-phenol dimer, monitoring the coherence dynamics in a closed- and open-system propagation varying the population time. Spectral signals are broadened using a Gaussian function.

### 4.1 Benzene

The benzene wave function was constructed by including 24 excited states within the energy range of 5.6–10.3 eV, because excited states with energy beyond this window cannot be excited by the pulses considered in the simulation. The delay time between the second and third pulses, *T*, was fixed at 100 fs and the temporal width of the pulses was δ = 2 fs. Two pulse setups were explicitly considered. In the first case, the incident frequency was chosen to be resonant with the excitation of the excited state at frequency ω = 5.61 eV (S_1_), which has the largest oscillator strength among the low-lying excitations (Table S1 of the Supporting Information). Additionally, simulations were performed with the incident frequency tuned close to the excitation energies of the fifth and sixth excited states included in the wave function expansion (ω = 7.33 eV, Table S1 of the Supporting Information). In both cases, three identical ultrashort laser pulses as in eq 4 have been employed. A ladder diagram illustrating the electronic states predominantly involved in the dynamics is shown in Figure 3a.

**Figure 3:**
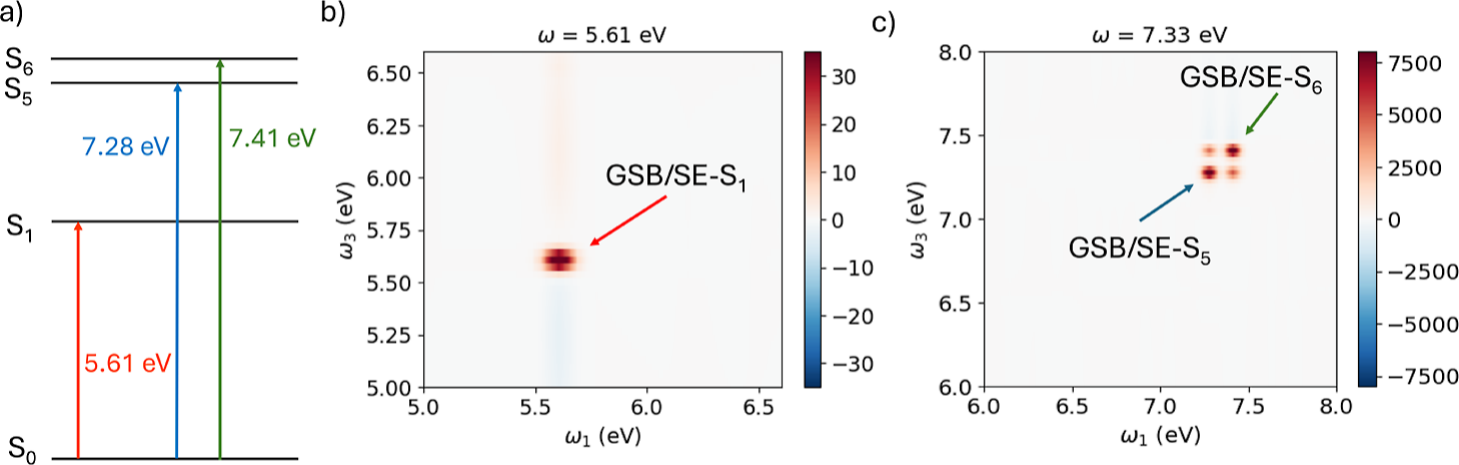
(a) Ladder scheme showing the benzene electronic transitions excited in the 2DES simulations. (b) 2DES absorptive map of benzene excited with pulse frequency centered at ω = 5.61 eV at *T* = 100 fs. (c) 2DES absorptive map of benzene excited with pulses frequency centered at ω = 7.33 eV at *T* = 100 fs. GSB/SE-S_*n*_ indicates that GSB and SE involve the excited state S_*n*_. Dynamics performed via eq 2.

The absorptive map for excitation at ω = 5.61 eV is shown in Figure 3b, while the corresponding map for excitation at ω = 7.33 eV, is reported in Figure 3c.

In Figure 3b, only a positive diagonal peak is observed, corresponding to incident and detection frequencies matching the bright electronic state of benzene at 5.61 eV. This signal corresponds to ground state bleaching (GSB) and stimulated emission (SE) which are typically superimposed. The first excited state, computed at the GW/BSE level, has an energy of 5.13 eV (*n* = 1 in Table S1 of the Supporting Information), which is in good agreement with the experimental value of 4.89 eV^71^ and also with the theoretical value computed at CASPT2 level of theory.^30^ However, this state is not visible in the 2DES map because the corresponding transition is weak. These findings agree with experimental observations,^71,72^ which report low-intensity bands near 5 eV and a pronounced absorption around 7 eV. The second dipole-allowed transition (*n* = 5, S_2_ in Table S1 of the Supporting Information) has an oscillator strength approximately 2 orders of magnitude smaller than that of S_1_, and is therefore not visible in the 2DES map. Higher-lying excitations fall outside the frequency range accessible by the electric field used in the real-time simulations.

By tuning the excitation frequency, different spectral regions become accessible in the 2DES maps. For instance, when pulses with an excitation frequency of ω = 7.33 eV are employed, states S_5_ (*n* = 17 in Table S1 of the Supporting Information) and S_6_ (*n* = 23 in Table S1 of the Supporting Information) are excited, as shown in Figure 3c. In this case, the 2DES map shows two positive diagonal peaks, corresponding to incident and detection frequencies equal to S_5_ and S_6_ excitation energies of benzene. A direct comparison of the simulated maps in Figure 3 with other theoretical studies cannot be easily done because the simulations employ different spectral ranges. For example, ref 20 considers excitation at 5.2 eV, and the 2DES maps in ref 30 cover energies below 5.2 eV, whereas the energy window in the present work is larger, ranging from 5 to 8 eV, as observed in Figure 3.

### 4.2 Chlorophyll *b*

The second system taken into account is the porphyrin core of chlorophyll *b*. The real-time dynamics simulations were performed by solving the TDSE. The system was excited using three identical ultrashort laser pulses, as described in eq 4, with a temporal width of δ = 5 fs, and pulses frequency ω = 2.31 eV resonant with the first excited state, corresponding to the *Q*_*y*_ band. The delay between the second and third pulses was fixed at 100 fs.

Figure 4 displays a ladder diagram that highlights the key electronic states of the system and the corresponding absorptive 2DES spectrum. In the 2DES map, a prominent positive diagonal peak is observed at pump and probe frequencies that matches the excitation energy of the first excited bright state (S_1_), which is resonant with the pulse frequency. This peak arises from ground-state bleaching (GSB) and stimulated emission (SE) contributions, and corresponds to excitation of the *Q*_*y*_ band^73^ of chlorophyll *b*. Apart from the excitation energy—which is overestimated by 0.4 eV with respect to the experimental value, likely due to different conditions (the experiment was performed in an EtOH/MeOH 4:1 mixture, while the calculations were carried out in vacuum) the computed 2DES spectrum closely resembles the experimental results.^73^

**Figure 4:**
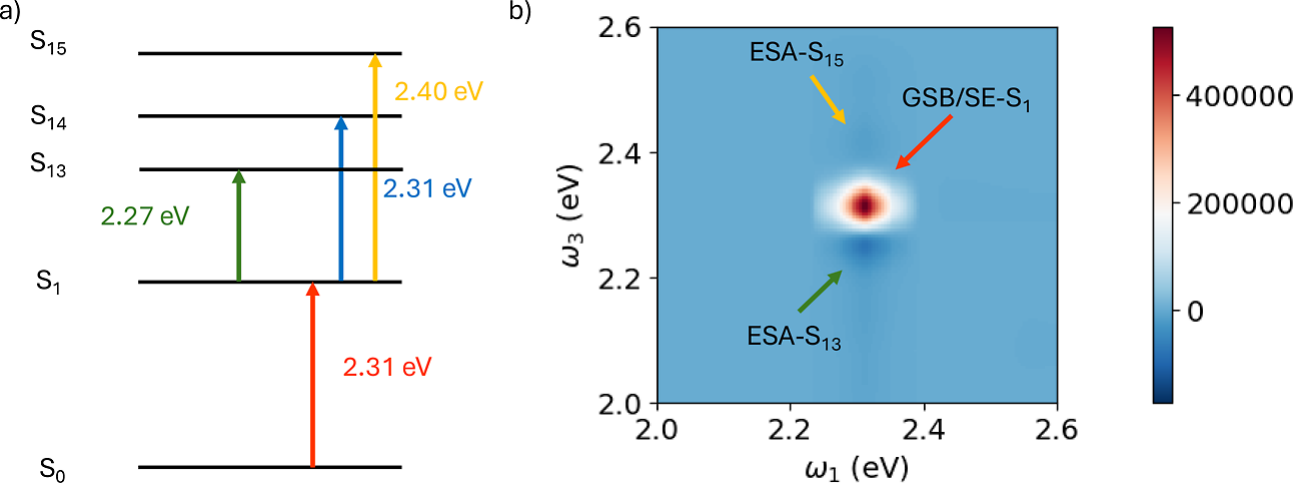
(a) Ladder scheme showing the most relevant electronic states for interpreting the 2DES spectrum. Only excitations S_1_, S_13_, S_14_ and S_15_ are shown. (b) 2DES absorptive map of the porphyrin core of chlorophyll *b*. GSB/SE-S_*n*_ indicates that GSB and SE involve the excited state S_*n*_, while ESA-S_*n*_ is for ESA from S_1_ to S_*n*_. Dynamics performed via eq 2.

Higher excited states cannot be directly accessed from the ground state because their energies lie outside the spectral bandwidth of the applied pulses. However, states with energies close to twice the pulse frequency can be reached via excited-state absorption (ESA) from the first excited state. In particular, ESA results in non-negligible population of states between S_13_ and S_15_. The extent to which these upper states are populated depends on both the spectral width of the pulses and the transition dipole moments between S_1_ and the higher excited states, which govern the transition strength.

A negative peak is observed on the 2DES map at the pump frequency corresponding to the excitation energy of S_1_ and the probe frequency that corresponds to the transition from S_1_ to S_13_ (2.27 eV), which is very close to the pulse frequency. The excitation of state S_14_ is not visible because of the superposition with the positive peak owing to SE and GSB of S_1_ in the same position. Additional weaker features (blue-shifted signals) are visible at the same pump frequency and probe frequencies are increasingly offset from 2.31 eV, which marks the center of the pulse spectrum.

### 4.3 Benzene-Phenol Dimer

The first 150 excited states, calculated at GW/BSE level, were used in the real-time dynamics simulations. The system was excited using three identical ultrashort laser pulses, as described in eq 4, each with a temporal width of δ = 2 fs and a central frequency of ω = 5.47 eV. The bandwidth of the incident pulse is broad enough to cover the excitation range of the first 20 excited states. The delay between the second and third pulses *T* was varied from 8.47 to 27.33 fs.

The molecular orbitals that contribute most to the excited states of the dimer with the highest oscillator strengths (states S_5_, S_13_, S_15_ and S_21_) are shown in Figures S1–S4 of the Supporting Information. Each excitation is a linear combination of transitions among multiple molecular orbitals, and the dominant contributing orbitals (those depicted in Figures S1–S4) are primarily localized on the phenol unit. An exception is the *S*_15_ excited state, whose leading contribution originates from an excitation on the benzene molecule.

Figure 5a shows the evolution of the 2DES map of benzene-phenol dimer at different population time *T*. The most intense diagonal peak corresponds to the excitation of the state at 5.76 eV, which is the bright state closest to the center of the pulse. Other excited states visible in the map are located at 4.96 eV (the first bright state), 5.93 and 6.05 eV. These diagonal peaks in the 2DES maps have positive sign and the intensity remains constant as a function of the population time, as expected for a closed-system dynamics. In contrast, the off-diagonal peaks arise from electronic coherences, which are already visible at *T* = 0 fs due to the simultaneous excitation of multiple states by the same pulse. Panel (b) of Figure 5 reports the evolution of the coherence between states S_5_ (4.96 eV) and S_13_ (5.76 eV) as a function of the population time, obtained by tracking the intensity of the peak at a pump frequency of 4.96 eV and a probe frequency of 5.76 eV. This coherence corresponds to the point highlighted with a black circle in the 2DES map. The time evolution of the coherence between states S_13_ (5.76 eV) and S_21_ (6.18 eV) is shown in Figure 5c, corresponding to the point on the map at a pump frequency of 5.76 eV and a probe frequency of 6.18 eV, highlighted with a red circle. As in the case of benzene-only, a direct comparison with the results in ref 30 is complicated by the different energy ranges analyzed.

**Figure 5:**
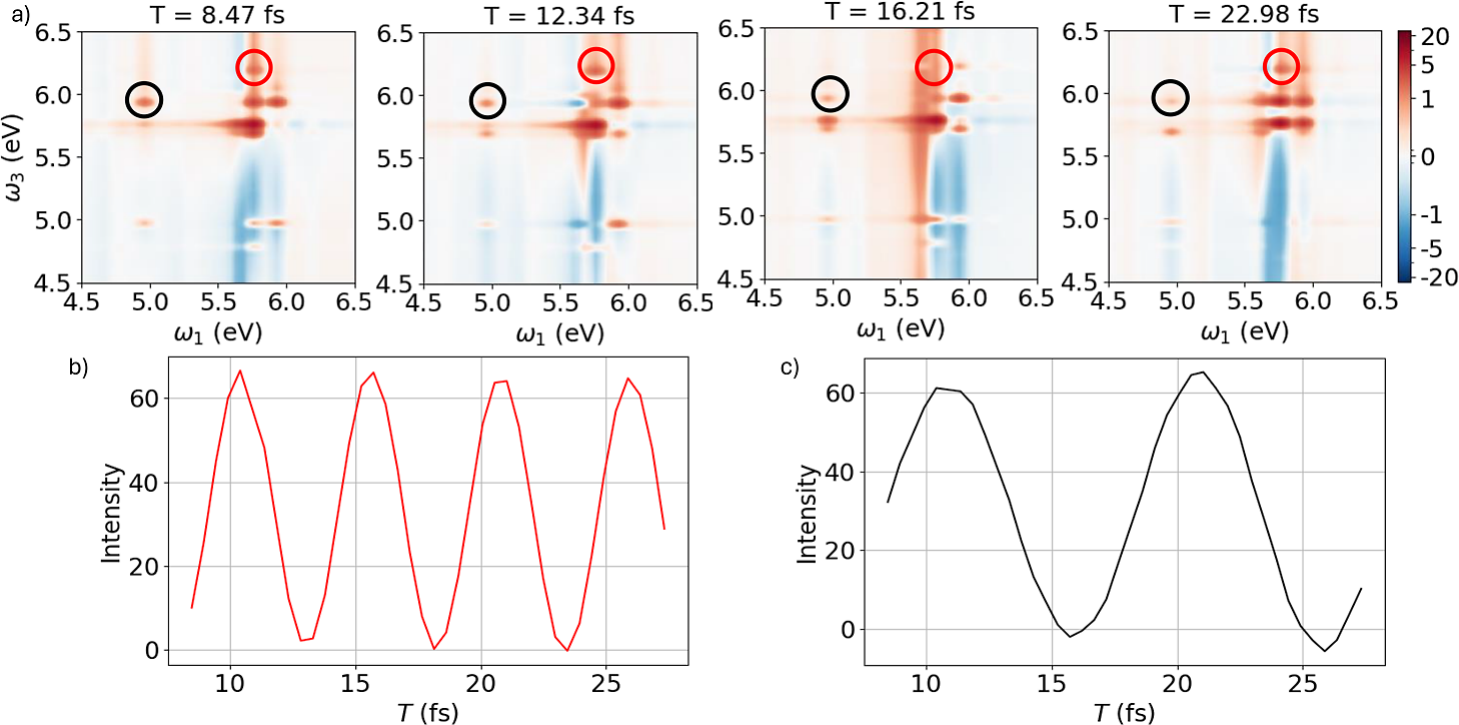
(a) 2DES map of benzene-phenol dimer at four different population times. The red and black circles represent the points of the 2DES maps shown in panels b and c. (b) Evolution of the coherence between states S_5_ and S_13_, corresponding to the red circles in the 2DES maps, as a function of the population time. (c) Evolution of the coherence between states S_13_ and S_21_, corresponding to the black circles in the 2DES maps, as a function of the population time. All the results have been obtained via eq 2.

Since decoherence processes were neglected in these simulations, the coherences do not decay as a function of population time. Both coherences oscillate over time with a frequency equal to the energy difference between the two states involved.

#### 4.3.1 Role of the Dephasing

Real-time dynamics of the dimer were performed including pure dephasing among all the coherences involved, with a fixed dephasing time of 60 fs, by means of SSE in eq 9. In other words, the environment effect is erasing electronic coherence: *M* coincides with the number of state pairs. In these simulations, the system was reduced to include only the five excited states with the largest oscillator strengths. Seventeen population times *T*, ranging from 5 to 95 fs, were considered, and 100 independent SSE trajectories were simulated for each value of *T*. The averaged 2DES maps were obtained from the ensemble-averaged dynamics of the dipole moment. As shown in the Supporting Information (Figure S5), SSE calculations with 100 trajectories for a simpler case (only one pulse, no phase cycling) are reasonably converged, though slightly more noisy than the time profile with 200 trajectories.

Figure 6 shows two representative 2DES maps, corresponding to population times of 24.99 and 74.94 fs. Two diagonal peaks are visible, associated with the excitation of the states at 5.76 and 5.93 eV. Although the diagonal peak at 4.96 eV is not clearly resolved, the coherence between the states at 4.96 and 5.76 eV can still be observed. Panel (b) of Figure 6 compares the evolution of the coherence between the states S_13_ and S_15_, respectively at 5.76 and 5.93 eV, as a function of the population time for two cases: dynamics performed including dephasing (as shown in panel a) of Figure 6), and dynamics where dephasing is neglected. The blue data points were fitted with a damped cosine function, yielding a dephasing lifetime of 50 ± 15 fs, while the orange data points were fitted with a simple cosine function. For ease of comparison, the amplitudes of the closed-system results were scaled by *a* factor of 1/10. At short population times, the coherence between the two states is maintained in the open-system dynamics; however, it progressively decays due to environmental interactions, approaching zero once the population time exceeds the dephasing time scale.

**Figure 6:**
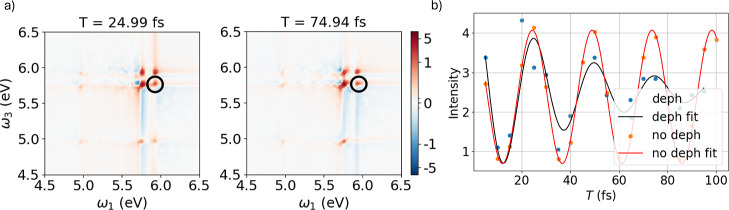
(a) 2DES maps of the benzene–phenol dimer at two different population times, obtained from SSE propagation with dephasing time *T*_2_ = 60 fs. (b) Evolution of the coherence between states S_13_ and S_15_, corresponding to the black circles in the 2DES maps. The blue points represent the peak intensity in the 2DES maps obtained from a dynamics with dephasing included (corresponding to the maps in panel (a)), while the black line shows the damped cosine function used to fit the data. The orange points and red line represent the corresponding signal intensity and fit obtained from a dynamics without dephasing. Results have been obtained via eq 2 (no dephasing) and 9 (dephasing with *T*_2_ = 60 fs).

## 5 Conclusions

In this work, we have presented a computational framework for simulating 2DES based on TDSE or SSE propagation. The main innovative aspects of this approach include: (i) the use of explicit wave function propagation as opposed to density matrix, which enables a direct interface with quantum chemistry outputs; (ii) the representation of the electronic states of molecular targets through the GW/BSE formalism; (iii) the incorporation of SSE to efficiently introduce environmental effects such as decoherence. The innovation brought with this approach allows to faithfully reproduce realistic experimental setups.

The proposed methodology was applied to three molecular systems: benzene, chlorophyll *b*, and the benzene–phenol dimer. For benzene, we explored the effect of varying laser pulse frequencies on 2DES maps at fixed population time in the absence of environmental interactions. The porphyrin core of chlorophyll *b* served as a prototype of a large molecular system relevant to energy transfer mechanisms. Its 2DES maps, obtained at fixed population time, clearly exhibit features such as SE and ESA. Finally, the benzene–phenol dimer was investigated to assess coherence dynamics in systems with many excited states, both in the absence and presence of pure dephasing effects.

The proposed method is extendable to include vibronic effects,^23^ following what has been done for studying decoherence in single molecules^48^ and (surface-enhanced) molecular Raman scattering.^74,75^ Other applications of this framework will focus on the simulation of 2DES maps of plasmonic systems,^76^ which can not be easily studied by wave function methods but are accessible to TDDFT and beyond-TDDFT approaches, such as GW/BSE.^42^ This will also include those systems operating under strong light–matter coupling conditions where plexcitonic states emerge.^77^ Extension to solid systems should also be feasible by carefully sampling the Brillouin zone.

## Supplementary Material



## Notes

Energies and transition dipole moments computed at GW/BSE level of benzene, chlorophyll *b* and benzene-phenol dimer. Cartesian coordinates of chlorophyll *b*. Involved molecular orbitals of the dimer. Convergence of SSE calculations (PDF)

## ⊥

Elettra Sincrotrone Trieste, SS 14 Km 163.5 in AREA Science Park, Basovizza, Trieste, Italy
