# Supplementary material for: Time-Dependent Open-Quantum Approach to Two-Dimensional Electronic Spectroscopy within a GW/BSE Active Space
Source: J Chem Theory Comput. 2026 Feb 25;22(5):2442–52. doi: 10.1021/acs.jctc.5c02002 (PMC12980717; doi:10.1021/acs.jctc.5c02002)
Supplement: Supplementary file 1 [file ct5c02002_si_001.pdf]

Supporting Information for "Time-dependent open-quantum approach to two-dimensional electronic spectroscopy within a GW/BSE active space"

Giulia Dall'Osto[a],*,† Margherita Marsili,‡ Stefano Corni,¶,§ and Emanuele Coccia*,†

†*Dipartimento di Scienze Chimiche e Farmaceutiche, University of Trieste, via L. Giorgieri 1, 34127, Trieste, Italy*

‡*Dipartimento di Fisica e Astronomia "Augusto Righi", University of Bologna, Viale Berti Pichat 6/2, 40127, Bologna, Italy*

¶*Dipartimento di Scienze Chimiche, Università di Padova, via F. Marzolo 1, 35131, Padova, Italy*

§*Istituto Nanoscienze-CNR, via Campi 213/A, 41125, Modena, Italy*

E-mail: giulia.dallosto@elettra.eu; ecoccia@units.it

[a]Present affiliation: Elettra Sincrotrone Trieste, SS 14 Km 163.5 in AREA Science Park, Basovizza, Trieste, Italy

Energies and Dipole moments of Benzene

Table S1: Energies and transition dipole moments from ground state to state state of benzene. n are label of all the states computed, λ are the label of the states included in the time-dependent calculations of the 2D spectra.

n	Energy (eV)	μ_x (a.u.)	μ_y (a.u.)	μ_z (a.u.)	λ
1	5.1261	0.000000	0.000000	0.000000	
2	5.1434	0.000000	0.000000	0.000164	
3	5.3049	0.000000	-0.009776	0.000000	
4	5.6069	0.000000	0.000000	-0.643835	S_1
5	5.6937	0.000000	0.000000	-0.080733	S_2
6	5.7442	0.000000	0.000000	0.000000	
7	5.8490	0.000000	0.000000	0.000000	
8	5.9735	-0.005961	0.000000	0.000000	
9	6.3230	0.000000	0.000000	0.000000	
10	6.3294	0.000000	0.000000	0.000129	
11	6.3690	0.000000	0.000000	0.000060	
12	6.3695	0.000000	0.000000	0.000000	
13	6.5179	-0.891199	0.000000	0.000000	S_3
14	6.5411	0.000000	-0.817207	0.000000	S_4
15	6.7464	0.000000	0.000000	0.000000	
16	6.7605	0.000000	0.000000	0.000147	
17	7.2784	-2.089893	0.000000	0.000000	S_5
18	7.2801	0.000000	0.000000	0.011154	
19	7.2827	0.000000	0.000265	0.000000	
20	7.2976	0.000000	0.000000	0.000000	
21	7.3493	0.000617	0.000000	0.000000	
22	7.3677	0.000674	0.000000	0.000000	

23	7.4128	0.000000	-2.116570	0.000000	S_6
24	7.4369	0.000000	0.000000	0.000000	
25	7.4451	0.000000	0.000384	0.000000	
26	7.5351	0.000000	0.000000	-0.154226	S_7
27	7.5488	0.000000	0.000000	-0.103905	S_8
28	7.5697	0.000000	0.000000	0.000000	
29	7.7177	0.000000	0.000000	0.000000	
30	7.7317	0.000000	0.000000	0.021249	
31	7.7437	0.000000	-0.000002	0.000000	
32	7.7504	0.000191	0.000000	0.000000	
33	8.0525	0.000000	0.000000	0.000000	
34	8.0670	0.000000	0.000000	0.246198	S_9
35	8.0677	0.000000	0.000000	-0.014408	
36	8.1701	0.000000	0.000000	-0.002835	
37	8.2095	0.000000	0.000000	0.000000	
38	8.2339	0.007201	0.000000	0.000000	
39	8.2580	0.000000	0.000000	0.000000	
40	8.3069	0.000000	-0.300357	0.000000	S_{10}
41	8.3212	0.356841	0.000000	0.000000	S_{11}
42	8.3339	0.000000	0.000000	-0.045448	
43	8.3607	0.000000	0.009239	0.000000	
44	8.3940	0.000000	0.000000	0.000000	
45	8.4316	0.000000	0.000000	0.000000	
46	8.4502	0.000000	0.000000	1.017812	S_{12}
47	8.4947	0.000000	0.000000	0.000154	
48	8.5487	0.000000	0.000000	0.000000	
49	8.5673	0.000000	0.000000	0.000577	

50	8.6116	0.000000	0.000000	0.000000	
51	8.6904	0.000000	0.000000	-0.623679	S_{13}
52	8.7795	0.000000	0.011256	0.000000	
53	8.8073	-0.008661	0.000000	0.000000	
54	8.8301	-0.000178	0.000000	0.000000	
55	8.8312	0.000000	0.000004	0.000000	
56	8.8544	0.000000	-0.000163	0.000000	
57	8.8581	-0.000254	0.000000	0.000000	
58	8.8616	-0.049367	0.000000	0.000000	
59	8.8731	0.000137	0.000000	0.000000	
60	8.8965	0.000000	-0.127725	0.000000	S_{14}
61	8.9951	0.000000	-0.000043	0.000000	
62	9.1170	0.000000	0.000000	0.000000	
63	9.1233	0.000000	0.000000	-0.000050	
64	9.1265	0.000000	0.000000	0.000000	
65	9.1873	0.000000	0.000000	0.009233	
66	9.1942	0.000000	0.000000	0.000000	
67	9.2314	0.000000	0.000000	-0.000062	
68	9.2569	0.000000	0.000000	0.000000	
69	9.2703	0.000000	0.000000	0.000073	
70	9.3102	0.000000	-0.000530	0.000000	
71	9.3183	0.000761	0.000000	0.000000	
72	9.4499	0.000000	0.000092	0.000000	
73	9.4538	-0.000124	0.000000	0.000000	
74	9.5025	0.000000	0.000000	0.002446	
75	9.5177	0.000000	0.000000	0.000000	
76	9.5444	0.000000	0.000000	0.000000	

77	9.5558	0.000000	0.000000	0.000048	
78	9.6056	0.000000	0.000000	0.000000	
79	9.8374	0.000000	0.000000	-0.004183	
80	9.8390	0.000000	0.000000	0.000000	
81	9.8461	0.708814	0.000000	0.000000	S_{15}
82	9.8523	0.000000	-0.685685	0.000000	S_{16}
83	9.8658	0.000000	0.000000	0.000000	
84	9.8993	0.000000	0.000000	0.000000	
85	9.9178	0.000000	0.000000	0.000015	
86	9.9658	0.000000	0.000135	0.000000	
87	9.9747	0.000000	0.000000	0.000000	
88	9.9846	0.000000	0.000000	-0.000029	
89	10.0667	0.000000	0.000000	-0.000001	
90	10.1087	-0.426597	0.000000	0.000000	S_{17}
91	10.1236	0.000000	-0.430982	0.000000	S_{18}
92	10.2469	0.000000	0.000000	-0.735822	S_{19}
93	10.2514	0.980873	0.000000	0.000000	S_{20}
94	10.2551	0.000000	-0.917355	0.000000	S_{21}
95	10.2701	-0.000002	0.121161	0.000000	S_{22}
96	10.2711	0.503443	0.000000	0.000000	S_{23}
97	10.2804	0.000000	-0.580878	0.000000	S_{24}
98	10.3313	0.000000	0.000000	-0.000202	
99	10.6728	0.000000	0.000424	0.000000	
100	10.6827	-0.000530	0.000000	0.000000	

Geometry, Energies and Dipoles of chlorophyll b

Table S2: Optimized geometry of the phorphirin core of chlorophyll b.

atom	X(Å)	Y(Å)	Z(Å)
C	0.10529334694944	-16.80590882000487	4.83148081667821
C	0.76060581551886	-16.25720935655461	6.01137134922189
N	2.00842155160294	-15.85980178891825	5.70997972327535
C	2.21528092975432	-16.12133791334643	4.36925061205200
C	1.00741724721432	-16.73222132537519	3.81837065283380
C	0.17219533719506	-16.16506981852268	7.27766927170053
C	0.72672937539966	-15.64415088504209	8.43009385576928
N	1.94931458272476	-15.09812920222679	8.55543203936852
C	2.12689911240555	-14.63388966512449	9.84237501879451
C	0.84429967745891	-14.74348953639265	10.63718269205772
C	-0.00895288186930	-15.66755860951192	9.75373290196410
C	3.31038406226112	-14.11758428945044	10.26873978630416
C	3.70654346847429	-13.58700176519109	11.64612462708964
C	5.17986669648649	-13.08062715150783	11.47879303159076
C	5.55107376854556	-13.35251584966810	10.09580014907703
C	4.45106182902826	-13.95434811453197	9.44677746178678
C	6.55620130898598	-13.22722555923439	9.16138333664618
C	6.00180058603909	-13.77516349264408	7.94935343992754
N	4.69828062217072	-14.21120031764822	8.17654078369003
C	3.59076029985799	-14.63793259411290	12.72977322824556
O	2.67528417505556	-14.71182417145092	13.50761477293534
O	5.80765558598602	-12.55681525150115	12.36565956147307
Mg	3.34457775877403	-15.03455152513453	6.95760120630170

N	4.74480078427591	-14.89185744026671	5.44690391295186
C	4.55308597228726	-15.27392115991657	4.17508946189924
C	5.73469153270925	-15.00790860218553	3.40394132696568
C	6.66177514962491	-14.45017339775366	4.26824707097466
C	6.02551353959751	-14.38154005801301	5.53985628064754
C	6.59803235852171	-13.87132647357698	6.70929741235899
C	7.91782338073153	-12.65358851115808	9.34277190277945
C	-0.11486210586204	-17.08661452887372	10.31056955857346
C	0.19724010469947	-13.37911230918209	10.91484168646848
C	-0.03135011964498	-12.52827981425161	9.67691674088548
C	-0.70549799315223	-11.22226627665925	9.98773133514200
O	-1.06624036873658	-10.85218423461384	11.07206235325590
C	3.35863445162283	-15.86181248376470	3.67700424592875
C	8.03900938070620	-13.96366991585370	3.94515805305967
C	8.06516476525499	-12.50775678178727	3.47478648337105
C	0.86865344717502	-17.13081697798214	2.42283802127930
C	0.14089710765616	-18.14574099984388	1.97356187146782
C	-1.30243564917180	-17.29172996323906	4.79623788239204
O	-0.87547457383672	-10.47223095521488	8.87666225071903
H	-1.31242392504015	-9.65387043265523	9.16190319374512
O	-0.87547457383672	-10.47223095521488	8.87666225071903
H	-1.31242392504015	-9.65387043265523	9.16190319374512
O	4.60974080166902	-15.50323969022205	12.69762581276967
C	4.57786798132766	-16.53116876658554	13.69622007707032
H	-0.83109866124987	-16.55537408927524	7.37153236999249
H	3.38937045852093	-16.12317175101720	2.63045709068080
H	7.61346154604464	-13.50602327782047	6.62870732965280

H	1.03711138323177	-15.20370441806303	11.60523262996338
H	-1.01706569315233	-15.26958067375070	9.62610735131135
H	-0.66896843890678	-17.73744162969394	9.63548124184372
H	0.87571178579333	-17.51908337146733	10.45706708356862
H	-0.62660727101602	-17.07781445671101	11.27278991643811
H	0.80843411829508	-12.82444877410560	11.62506373986102
H	-0.75461541845278	-13.54810820328255	11.41950431682040
H	-0.64837063794584	-13.03707375072304	8.93318593045937
H	0.90746755318623	-12.29708439499429	9.16987554373368
H	-1.94286029725302	-16.68746733614810	5.43786401335155
H	-1.69961834553759	-17.24679700752171	3.78404010572583
H	-1.38243943199310	-18.32672100452611	5.13662619578596
H	8.48049098730079	-14.59814133132916	3.17683710249087
H	8.67846565001168	-14.07305659097559	4.82139167506767
H	9.08554303269329	-12.19185391463087	3.25891391482367
H	7.47139684642944	-12.37960464143922	2.57027151373911
H	7.65572842434884	-11.84315323722750	4.23520099938989
H	8.09055708135069	-11.82035288968925	8.66003367981431
H	8.04361794950890	-12.29301544990049	10.36044497802670
H	8.68851056150581	-13.40123571679654	9.14837704389152
H	3.09613356060823	-12.74549048327337	11.97041243189306
H	1.43382579785293	-16.54748623758357	1.70440535728754
H	0.09899572845792	-18.36796620097229	0.91627155233005
H	-0.40609435704613	-18.80187787967853	2.63620848742577
H	5.47458724556126	-17.12032183909715	13.53820609720104
H	4.57955541981779	-16.08825384471540	14.68916224924530
H	3.68820288897115	-17.14586599421373	13.57989050739187

C	5.94511947116458	-15.26017076686292	1.98910234202376
O	5.12768065466408	-15.72098188853005	1.21752615712544
H	6.95129012879958	-14.99763017728868	1.61446179815412

Table S3: Energies and transition dipole moments from ground state to state λ of chlorophyll b.

λ	Energy (eV)	μ_x (a.u.)	μ_y (a.u.)	μ_z (a.u.)
1	2.27191	-0.83942090	-0.67029562	-1.26548681
2	2.63890	0.22201750	0.16037070	0.16720757
3	3.31365	-2.30624632	-0.22452618	2.63119143
4	3.47689	-1.49786694	-1.23753491	-2.58434465
5	3.59685	0.41256873	-0.14959441	-0.87468523
6	3.83286	-0.01762355	0.05152711	0.05340340
7	3.85531	0.12936982	0.08216821	-0.10162678
8	4.00154	-0.07376169	0.09628038	0.54051192
9	4.27847	0.92934024	0.28666150	-0.35747063
10	4.49198	0.72345764	0.04654356	-0.34535445
11	4.54959	0.19230037	0.33687086	0.90678891
12	4.63450	1.34913319	0.60582467	0.49736237
13	4.66344	-0.30685640	-0.39735609	-0.94212987
14	4.77463	0.01994922	0.01287264	0.03215255
15	4.80940	-0.05498257	-0.06821796	-0.31371502
16	4.95888	-0.55347884	-0.33291678	-0.35164946
17	4.96769	-0.11475598	-0.07472082	0.05529182
18	4.98734	0.46085615	0.30591026	0.45341175
19	5.15603	0.28255555	0.21865706	0.09610092
20	5.16242	-0.66517391	-0.19409230	0.35175544

Molecular orbitals of benzene-phenol dimer

The molecular orbitals involved in the excitations with the highest oscillator strength (states 5, 13, 15, 21) are reported in Figures S1-S4.

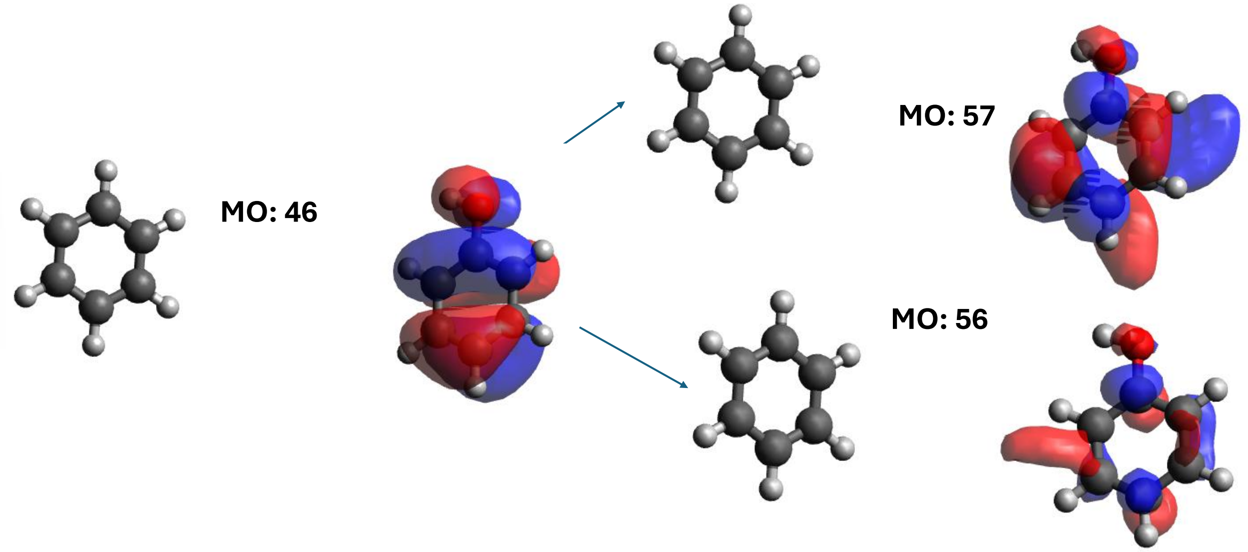

Figure S1: Molecular orbitals involved in the transitions that contribute to the 5th excited state of the benzene-phenol dimer.

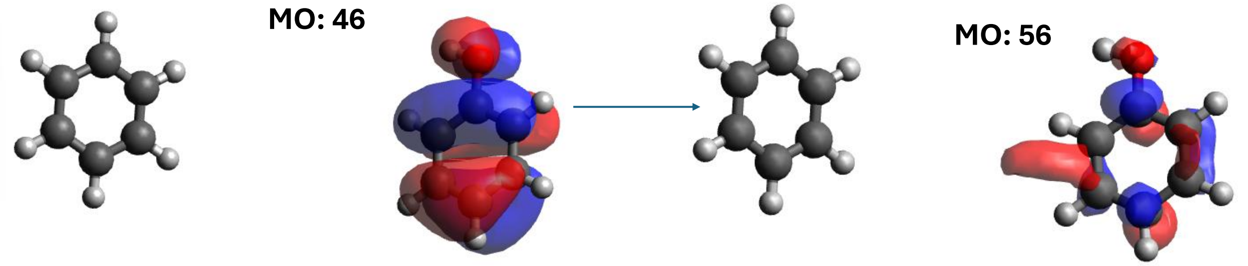

Figure S2: Molecular orbitals involved in the transitions that contribute to the 13th excited state of the benzene-phenol dimer.

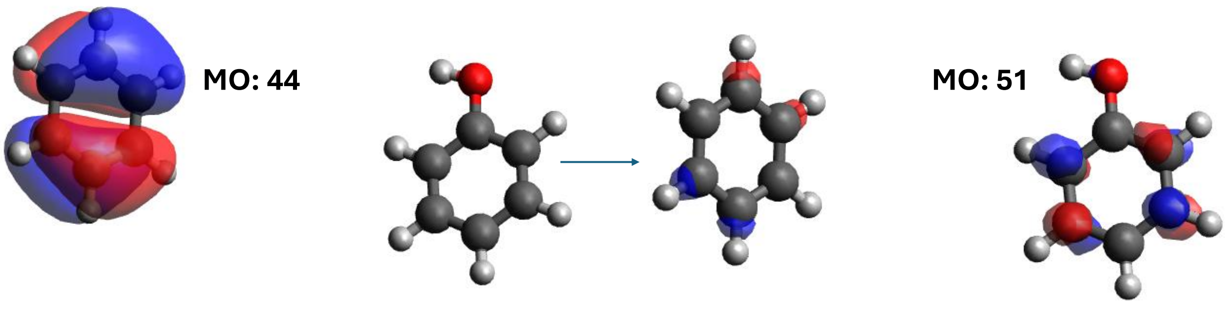

Figure S3: Molecular orbitals involved in the transitions that contribute to the 15th excited state of the benzene-phenol dimer.

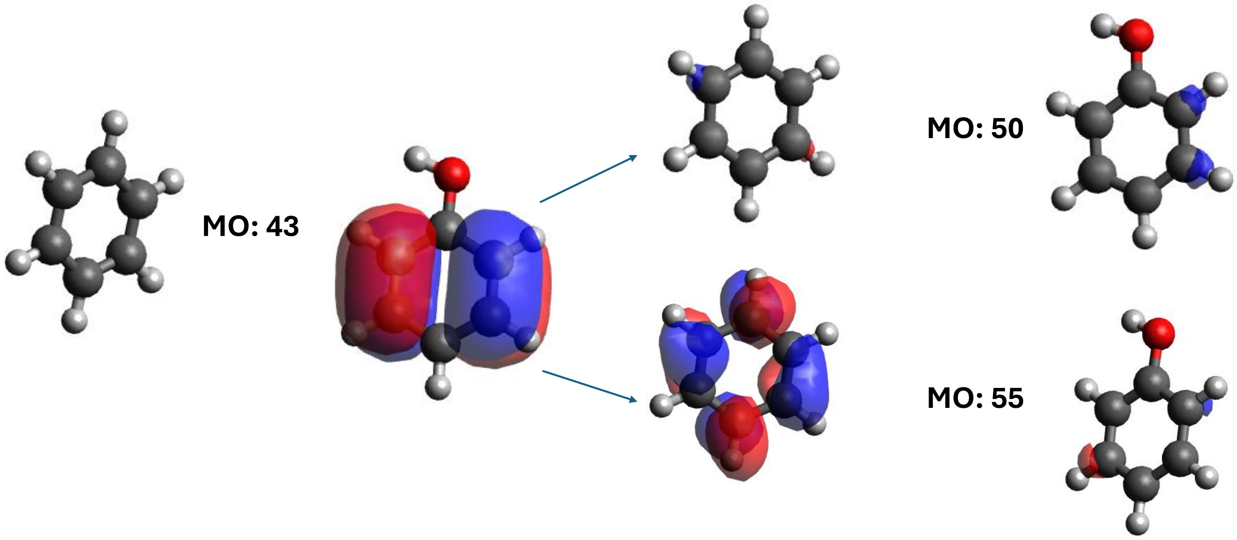

Figure S4: Molecular orbitals involved in the transitions that contribute to the 21th excited state of the benzene-phenol dimer.

Energies and Dipole moments of benzene-phenol dimer

Table S4: Energies and transition dipole moments from ground state to state λ of benzene-phenol dimer.

λ	Energy (eV)	μ_x (a.u.)	μ_y (a.u.)	μ_z (a.u.)
1	4.1248	-0.020173	0.057592	-0.010458
2	4.3048	-0.091042	0.118039	-0.129094
3	4.6797	0.101423	0.117018	0.085007
4	4.7847	0.158359	0.298608	0.039285
5	4.9744	0.973451	-0.042681	-0.320396
6	5.2293	0.060463	0.074250	-0.066217
7	5.2301	-0.027833	0.007069	-0.017220
8	5.2319	-0.022594	0.008571	0.004851
9	5.3102	-0.120438	0.058138	0.086143
10	5.6501	0.072172	-0.052364	-0.114348
11	5.6922	-0.073643	-0.183002	-0.160540
12	5.7314	-0.501971	0.315821	0.149648
13	5.7630	-0.346037	-0.239456	-0.058067
14	5.8342	0.432236	-0.018384	-0.295094
15	5.9358	0.097666	0.067483	0.585165
16	5.9646	-0.060846	0.135893	-0.234415
17	6.0273	-0.096358	-0.178889	-0.113672
18	6.0345	-0.076399	0.101541	0.022394
19	6.0728	-0.134374	1.051139	-0.742558
20	6.1616	0.032933	-0.122774	0.080673
21	6.1665	-0.522226	0.662365	-0.940818
22	6.4537	0.339394	0.238713	-0.330908

23	6.5025	-0.712207	-0.200219	0.466660
24	6.5165	0.859776	0.824874	-0.044424
25	6.5265	0.799060	-0.775783	-0.038268
26	6.5546	-0.194999	0.135871	-0.159856
27	6.5569	-0.006333	-0.011049	0.130326
28	6.5900	0.026256	0.000971	0.022424
29	6.6003	0.034836	0.077859	-0.027113
30	6.6672	-1.363894	0.073945	0.325057
31	6.7015	0.002804	0.039421	0.033374
32	6.7201	-0.040015	-0.015649	-0.009626
33	6.7234	0.568700	-0.009672	-0.132512
34	6.7752	-0.032791	-0.088074	-0.016675
35	6.7766	0.015367	-0.076875	-0.028403
36	6.7796	-0.270338	0.291095	-0.257308
37	6.8465	-0.214307	-0.091782	0.146136
38	6.8762	-0.271318	-0.141331	0.181689
39	6.9152	-0.205778	0.237463	-0.122681
40	6.9308	0.033123	-0.054885	0.060691
41	6.9945	-0.156988	0.013884	0.004867
42	6.9980	-0.014565	-0.129552	-0.140896
43	7.0478	0.209937	0.294369	-0.200148
44	7.1489	0.034667	0.131234	-0.229419
45	7.1804	-0.668626	-1.724782	0.001103
46	7.2136	1.725306	-0.669353	0.005738
47	7.2262	0.060091	0.000296	-0.197922
48	7.2609	-0.037628	-0.051817	-0.152101
49	7.3561	-0.091066	-0.179110	0.088856

50	7.3642	-0.002465	0.021353	-0.062697
51	7.3693	0.080722	-0.017609	-0.026635
52	7.3970	0.023932	0.116406	-0.005553
53	7.4085	0.013725	-0.013428	-0.022781
54	7.4193	0.062428	0.030852	0.009846
55	7.4320	-0.150726	-0.156177	0.076092
56	7.4414	-0.059134	0.045509	0.099599
57	7.4488	0.150365	-0.196015	0.056951
58	7.4731	-0.071124	-0.234524	-0.102272
59	7.4915	-0.001785	0.026164	-0.013688
60	7.4990	-0.084881	-0.064301	0.160882
61	7.5195	-0.021450	0.207459	0.200263
62	7.5382	-0.014887	-0.077416	-0.134673
63	7.5585	-0.012098	0.031165	0.015809
64	7.5658	0.101307	-0.184200	0.248634
65	7.5920	0.059045	-0.176985	0.060886
66	7.6421	0.009642	-0.020402	0.015931
67	7.6473	-0.028953	-0.017008	-0.117899
68	7.6530	0.125882	-0.065327	0.012091
69	7.7061	-0.060849	-0.045950	-0.154025
70	7.7562	0.056313	-0.098000	0.088299
71	7.7609	-0.053178	-0.071324	0.032606
72	7.7673	0.008174	0.116488	0.120636
73	7.7765	-0.104607	-0.059326	-0.025406
74	7.8053	-0.077353	-0.106198	0.014022
75	7.8316	0.076396	0.073078	-0.018474
76	7.8503	0.006133	0.033780	0.003943

77	7.8579	-0.002922	-0.008388	0.000101
78	7.8761	-0.039639	-0.043936	-0.037938
79	7.9018	-0.003216	-0.001069	-0.006788
80	7.9028	-0.013595	0.000289	-0.000813
81	7.9122	0.044918	-0.053828	-0.008004
82	7.9596	0.045612	-0.034099	0.086014
83	7.9781	-0.009300	0.096430	-0.194413
84	7.9886	0.233869	-0.251016	0.086053
85	8.0130	-0.073642	-0.119585	-0.137554
86	8.0288	0.006042	-0.081416	0.109983
87	8.0413	0.061595	0.019670	0.034761
88	8.0599	-0.061909	0.020766	0.008868
89	8.0813	-0.007670	-0.108469	0.030577
90	8.0844	-0.116718	0.063486	-0.056288
91	8.1063	0.000202	0.026494	-0.176968
92	8.1389	0.058368	0.020458	0.003390
93	8.1598	0.122743	-0.526151	0.631186
94	8.2135	-0.191263	-0.246182	-0.064850
95	8.2275	0.038269	-0.178824	0.047440
96	8.2453	-0.119098	0.141437	0.107523
97	8.2564	-0.021506	-0.027685	-0.077571
98	8.2592	-0.651131	0.043682	0.274004
99	8.3035	0.048962	-0.026289	0.072533
100	8.3201	-0.022976	0.006338	0.011427
101	8.3231	-0.018363	0.187947	-0.137152
102	8.3636	-0.399426	-0.026400	0.060226
103	8.4009	0.028145	0.057284	0.064370

104	8.4114	0.008962	-0.010573	0.004588
105	8.4140	-0.002414	-0.010607	0.204446
106	8.4252	-0.004198	0.008314	0.021040
107	8.4300	0.003090	-0.050155	-0.019686
108	8.4353	-0.011062	0.023895	-0.133693
109	8.4445	-0.014063	0.006124	-0.029362
110	8.4539	0.345781	0.022897	0.013981
111	8.4696	0.156614	0.392085	0.195287
112	8.4722	0.041370	0.084161	0.698337
113	8.4933	-0.029521	-0.001610	0.017164
114	8.5128	-0.104364	-0.245678	-0.071486
115	8.5216	0.051961	0.005066	-0.188263
116	8.5399	-0.043296	0.051314	-0.686543
117	8.5532	-0.189757	0.024564	0.102414
118	8.6133	0.054925	-0.181027	-0.251112
119	8.6171	-0.039437	-0.330815	-0.265838
120	8.6207	-0.137106	-0.087119	-0.016803
121	8.6259	-0.003434	0.088962	0.130068
122	8.6397	0.056403	-0.363944	0.084823
123	8.6515	0.246800	0.179965	-0.124924
124	8.6518	-0.342292	0.160253	0.017799
125	8.6639	-0.147997	0.083784	-0.112250
126	8.6646	-0.013797	-0.043650	0.014298
127	8.6938	-0.002757	0.047846	0.101698
128	8.6992	-0.006485	-0.015938	-0.019301
129	8.7169	-0.028070	-0.018962	-0.066408
130	8.7301	0.072053	0.026781	0.168282

131	8.7331	-0.032039	-0.192542	-0.127043
132	8.7493	0.027713	-0.015075	-0.373649
133	8.7505	-0.009527	-0.029645	0.067844
134	8.7605	-0.004017	-0.073914	-0.271462
135	8.7643	0.003641	0.094784	-0.268333
136	8.8028	0.014759	-0.017619	0.030368
137	8.8099	0.056289	-0.010844	-0.128841
138	8.8117	0.040464	0.057389	0.003026
139	8.8442	-0.050378	0.030196	-0.038199
140	8.8683	0.031155	-0.021015	-0.012042
141	8.8910	-0.003035	0.005719	0.013264
142	8.9035	0.040786	0.071008	-0.018607
143	8.9115	0.006275	-0.002268	-0.024830
144	8.9140	-0.005947	-0.018307	-0.030972
145	8.9616	-0.031050	0.013812	-0.009714
146	8.9697	-0.001574	-0.013942	0.031894
147	8.9736	-0.009814	-0.000844	0.001215
148	8.9859	-0.182682	-0.183960	0.037521
149	9.0018	-0.018812	-0.011157	0.066602
150	9.0214	0.006104	-0.081897	-0.253181

Convergence of the number of SSE trajectories

We compared the time evolution of two benzene–phenol dimer coherences, corresponding to the pairs of states (12–15) and (13–15), to assess whether the number of trajectories considered is sufficient to accurately describe the system dynamics. These simulations were performed including the five excited states of the benzene–phenol system used in the SSE dynamics reported in the main text (Section 4.3.1). The system was excited by a single laser pulse with the same temporal profile as that described in the main text (intensity of $I = 5.01 \times 10^9$ W/cm^2, temporal width of $\delta = 2$ fs and a central frequency of $\omega = 5.47$ eV).

The results obtained by averaging over 100 and 200 trajectories are shown in Figure S5. In particular, the comparison between panels (b) and (d) shows that the time evolution of the coherences until 100 fs after interaction with the incident pulse is nearly identical in both cases, confirming that the use of 100 independent SSE trajectories provides a reliable statistical sampling of the system dynamics.

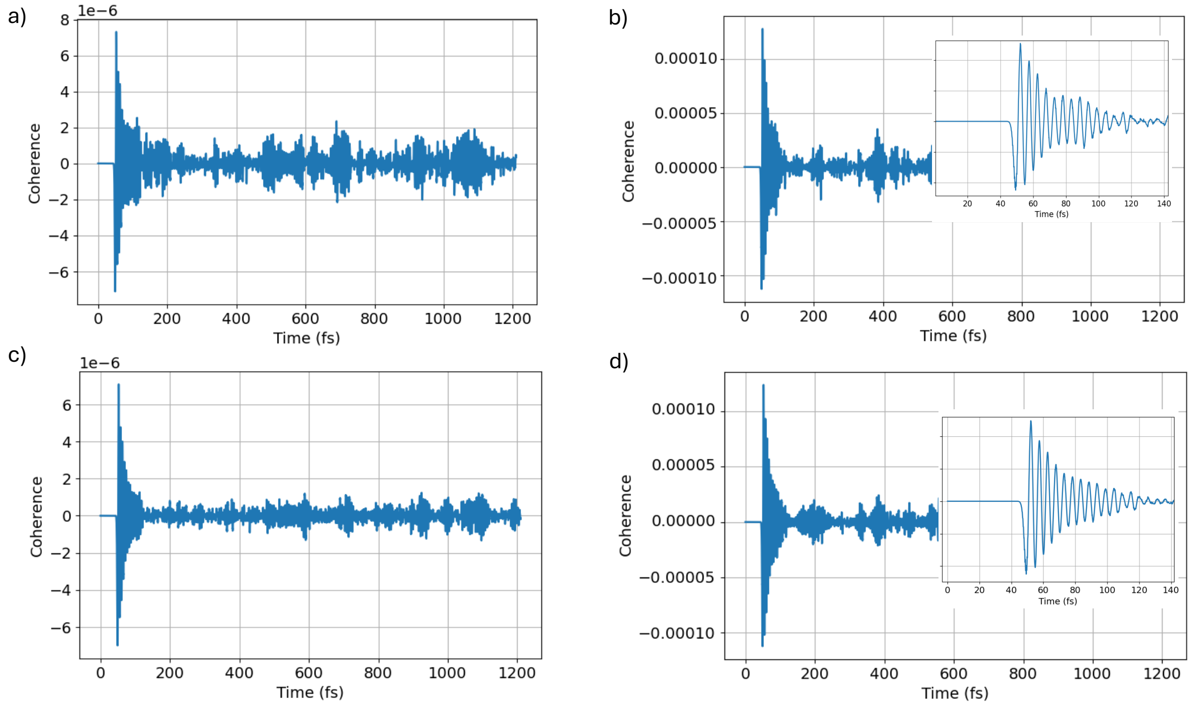

Figure S5: Evolution of the coherences as a function of time, when the system is interacting with a single pulse and pure electronic dephasing is included. a) Coherence between states 12-15, averaged over 100 trajectories. b) Coherence between states 13-15, averaged over 100 trajectories. c) Coherence between states 12-15, averaged over 200 trajectories. d) Coherence between states 13-15, averaged over 200 trajectories. In panel b) and b) the insight shows a magnification of the first 100 fs after the interaction with the pulse.
